# Superhelical Duplex Destabilization and the Recombination Position Effect

**DOI:** 10.1371/journal.pone.0020798

**Published:** 2011-06-09

**Authors:** Cheryl L. Sershen, Joshua C. Mell, Sally M. Madden, Craig J. Benham

**Affiliations:** 1 Baylor College of Medicine, Houston, Texas, United States of America; 2 Department of Zoology, University of British Columbia, Vancouver, British Columbia, Canada; 3 U.C. Davis Genome Center, Davis, California, United States of America; 4 Department of Mathematics and Genome Center, University of California, Davis, Davis, California, United States of America; Tulane University Health Sciences Center, United States of America

## Abstract

The susceptibility to recombination of a plasmid inserted into a chromosome varies with its genomic position. This recombination position effect is known to correlate with the average G+C content of the flanking sequences. Here we propose that this effect could be mediated by changes in the susceptibility to superhelical duplex destabilization that would occur. We use standard nonparametric statistical tests, regression analysis and principal component analysis to identify statistically significant differences in the destabilization profiles calculated for the plasmid in different contexts, and correlate the results with their measured recombination rates. We show that the flanking sequences significantly affect the free energy of denaturation at specific sites interior to the plasmid. These changes correlate well with experimentally measured variations of the recombination rates within the plasmid. This correlation of recombination rate with superhelical destabilization properties of the inserted plasmid DNA is stronger than that with average G+C content of the flanking sequences. This model suggests a possible mechanism by which flanking sequence base composition, which is not itself a context-dependent attribute, can affect recombination rates at positions within the plasmid.

## Introduction

When a reporter plasmid was placed at different locations in *Saccharomyces cerevisiae* chromosome III, the frequency of its experiencing double strand break (DSB) formation and its recombination rate were found to depend upon its genomic context [1]. An up to 10-fold variation in recombination rates was observed, depending upon where in the chromosome the plasmid was inserted. Moreover, gene conversion frequencies were seen to be tightly correlated with DSB formation rates within these heteroallelic constructs. These phenomena have come to be known as the recombination position effect. As the plasmid is 8 kb long and these recombination events occur in its interior, the attributes of its insertion site that affect its recombination rate must propagate over kilobase distances.

Subsequent work showed that the recombination rates observed for this heteroallelic plasmid when placed at different sites on chromosome III are positively correlated with the G+C-richness of the regions flanking the insertion site [2]. This suggests that the recombination position effect is both context-dependent and sequence-dependent. Some attribute associated with G+C-richness appears to propagate through the plasmid insert, affecting recombination frequencies at locations that are several kilobases within it. Several mechanisms have been proposed to explain this context-dependent effect. These include chromatin modifications associated with high GC content, a GC mutational bias in regions of high recombination, and energy constraints imposed by flanking sequences [1]–[3]. As both DSB formation and recombination rates may be influenced by changes in the stability of the DNA duplex, we consider the possibility that the G+C-richness of the regions flanking the plasmid insert may affect the rates of these processes in its interior through its effect on superhelical duplex destabilization.

DNA within living systems is topologically constrained so that varying levels of superhelicity can be imposed, either through enzymatic activity, the release or binding of architectural proteins and nucleosomes, or as a result of transcriptional activity [4]–[6]. Superhelicity topologically constrains the DNA experiencing it, and can drastically alter its duplex stability in a highly coupled, context-dependent manner [7], [8].

The stress-induced duplex destabilization (SIDD) method was developed to analyze the thermodynamic stability of DNA sequences under superhelical constraints, as occur *in vivo* [9]. This method uses a statistical mechanical, Ising model framework to analyze the transition properties of a user-specified sequence on which a user-specified superhelix density has been imposed. It calculates the destabilization (free) energy 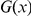, which is the incremental free energy needed to fully open base pair 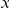, and the equilibrium probability of denaturation 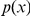 [10], [11]. This is done for each base pair in the sequence. The graph of the destabilization energy 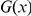 versus position 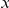 is the SIDD profile of the sequence under these conditions.

Because the susceptibility to duplex destabilization is strongly context-dependent, it can vary within an inserted sequence depending on the location where it is placed. The SIDD analysis predicts that under normal physiological conditions most base pairs in a negatively superhelical DNA remain stable, while a small fraction of base pairs become highly destabilized. Although the SIDD method has no tunable parameters, its predictions of both the locations and extents of these so-called stress-induced duplex destabilized (SIDD) sites have been shown to agree with experiments in every case where experimental information was available [9], [12]–[14]. This precision allows the SIDD methods to be used with confidence to analyze other sequences, on which experiments have not been performed.

Sites that are predicted to be destabilized by superhelical stresses have been shown not to occur at random within genomic sequences. Instead they are associated with specific regulatory regions, including transcription termination sites and promoters in prokaryotes, replication origins and eukaryotic scaffold/matrix attachment regions [8], [14]–[18]. Stress-regulated destabilization also has been shown to be an essential participant in the mechanisms by which specific transcriptional events are controlled [12], [19], [20]. SIDD analyses have illuminated a variety of normal and pathological biological phenomena [15], [16]. They also have provided a first insight into an important new class of mechanisms by which information may be transmitted along a DNA molecule through the global coupling exerted by imposed superhelicity [19], [21].

Although most eukaryotes do not have negatively supercoiling gyrases, recent experiments have shown that their genomic DNA experiences substantial levels of transcription-driven superhelicity [10]. Although transient, this superhelicity propagates over kilobase distances and is introduced substantially faster than topoisomerase enzymes act to relax it. In particular, it was shown that transcription-driven superhelicity in humans persists in a kinetic sense long enough to drive structural transitions at kilobase distances from the transcription event that causes it.

In this paper we investigate whether the differences in the superhelically induced duplex destabilization (SIDD) properties that occur when a plasmid sequence is placed in different contexts might explain the recombination position effect. This requires us to determine whether the SIDD profiles of the plasmid show statistically significant differences when the plasmid is located at different genomic positions. Unfortunately, to date there are no established procedures for performing statistically rigorous comparisons of closely related genomic profiles such as those produced by SIDD analysis. While pattern recognition algorithms have long been a topic of research in bioinformatics, to date they have not been developed for this purpose [22]. So in this paper we construct rigorous statistical methods to assess whether two profiles for a DNA sequence are significantly different. Although we do this specifically for SIDD profiles, the methods we use also can be applied to other types of sequence profiles.

In this paper we first analyze whether there are statistically significant differences in the superhelically induced duplex destabilization (SIDD) profiles of the plasmid when placed at the various locations identified in the original experiments on the recombination position effect in yeast. We then assess how well these differences correlate with the recombination rates occurring at these sites. We also assess how well the GC content of the flanks correlates with both recombination rate and with SIDD profile changes.

## Materials and Methods

To assess how SIDD property differences correlate with the recombination position effect seen in the experiments by Borde et al. [1], we constructed nine sequences corresponding to their plasmid inserts. This was done by placing the pmj115 plasmid, which is 8,560 base pairs long and contains the URA3 gene and an ARG4 fragment, into nine different locations in the *Saccharomyces cerevisiae* chromosome III. The resulting sequences were each 18,560 base pairs long. These nine constructs are named according to the locations of their insertion sites as YCL011C, YCR004C, YCR009C, YCR017C, YCR026C, YCR028C, YHR025W, YHR037W and YHR201C. These nine cases are the ones where we could determine the exact location of the insertion. The recombination rates for each of these inserts at their genomic positions were determined in the original paper [1]. For six of these nine inserts the DSB formation rates also were measured.

We used the WebSIDD algorithm to calculate the SIDD profile of each of these nine sequences, and of the plasmid alone, without flanks [9], [11]. This identified the sites within each sequence that are most susceptible to destabilization by superhelical stresses, and determined their relative susceptibilities. Because many circumstances can affect the overall level of unrestrained DNA superhelicity, it is not entirely clear what conditions best mimic the *in vivo* biological state. So in our calculations we imposed the conditions that we have found best identify SIDD sites. These are temperature 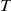 = 310K, 0.01 M salt concentration, and superhelix density 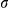  = −0.055.

We then applied a variety of statistical tests to determine how the recombination rate occurring in the plasmid when inserted at each of these nine locations correlates with changes in the superhelical stress profiles of the inserts. We also determined how well these recombination rates correlate with DSB formation and with the G+C content of the flanks, and how this G+C content correlates with SIDD properties. These methods and their results will be described in the next section.

## Results

### Superhelical Destabilization Profiles

**Figure 1:**
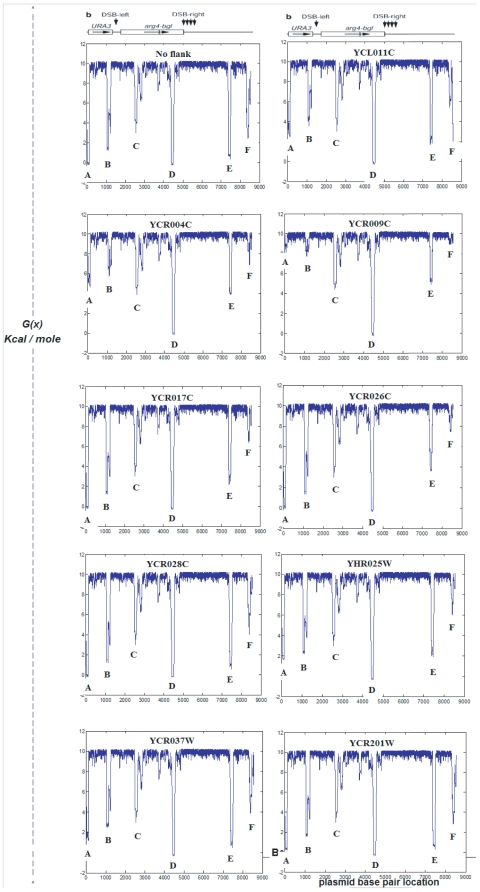
The SIDD profiles of the plasmid pmj115 alone, and when inserted in nine different genomic contexts. Only the profile of the plasmid itself is shown here; 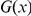 is measured in units of kcal/mole.

Figure 1 displays the ten calculated profiles for the plasmid region of each sequence. One sees that in each case the majority of the plasmid sequence remains stable (i.e. with high values of the destabilization energy 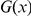.) Significant destabilization occurs only at six distinct “valleys”, marked A-F, which have low destabilization energies under these conditions.

Differences among these profiles are concentrated at these six destabilized regions. As the sequence itself is identical in each case, this effect must be due to the genomic context of each insert. This shows that differences in the base composition of the surrounding 5 kb flanks can indeed affect the destabilization properties of sites interior to the plasmid. Any change in the free energy required to denature a region has an exponentially magnified effect on the ease of opening of that region [8]. For example, a decrease in 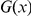 of 3 kcal/mol at a site increases its equilibrium probability of opening by two orders of magnitude. So relatively small differences among the destabilization profiles of these insert sequences can have significant effects on any process that is affected by strand separation.

The differences among these SIDD profiles may be quantified in several ways. One might compare the width of each valley between the profiles. Alternatively, one might compare their areas (in base pairs x energy) or their depths (in energy units). One might consider any of these measures in aggregate by summing over the entire profile. We used two statistical techniques to determine whether there are significant differences among the ten profiles as measured in each of these ways. These are the Komogorov-Smirnov (K-S) test and the Wilcoxon Rank Sum (equivalent to the Mann-Whitney U) test [23]. The main difference between these tests is in their null hypotheses. The K-S test has the null hypothesis that the empirical distributions are the same, while the Wilcoxon Rank Sum test postulates that the medians of the distributions are equal. Both tests, being non-parametric, make no *a priori* assumptions about the distribution of the data. However, they do assume that the samples are from independent and identically distributed random variables. This is not the case for destabilization within a single plasmid insert, as superhelicity couples together the behaviors of all base pairs that experience it. However, it is a reasonable assumption for comparisons between plasmids.

We used these tests to compare the destabilization energy (SIDD) profiles of the whole sequence (plasmid plus flanks), for the plasmid regions alone, and for each of the individual valleys A-F. The distributions of the each of the nine inserts with flanking regions (labeled whole sequence) were compared pair-wise, for a total of 36 comparisons. All of these were seen to differ significantly by the K-S test, and 32 of 36 were significant by the Rank sum test. There are a total of 45 pair-wise comparisons of the distributions across the plasmid only, due to the inclusion of the plasmid with no flanking regions. For each of the six valleys there also were 45 pair-wise comparisons.

The results of these pair-wise comparisons are summarized in Table 1, which shows the number of tests that found statistically significant differences at the 5% level. Of the 351 pairwise comparisons made, 286 are found by the K-S test to be significant at that level. This test is commonly regarded as being conservative, if anything tending to underrepresent the significance of its comparisons. Although in this situation the Rank Sum test is somewhat more conservative than the K-S test, it still finds significant differences in 62% of the pairwise comparisons. In both tests fewer significant differences between profiles are found at the central valleys, C and D, while the valleys nearer the edge of the plasmid are much more likely to differ between plasmids. This is not surprising as the influence of the flanking sequences on this transition may be expected to diminish with distance. Still, the K-S test finds approximately 40% of the comparisons involving the central valleys C and D to show significant differences at the 5% level.

**Table 1:** Results of Kolmogorov-Smirnov and Wicoxon Rank Sum tests, at the 5% significance level, using the pair-wise comparisons of the distribution of the 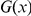 energies.

		K-S tests			Wilcoxon Rank sum		
T = 310,	Total						
sd = 0.055	Comparisions	significant	not significant	% significant	significant	not significant	% significant
whole sequence	36	36	0	100.0%	32	4	88.9%
whole plasmid	45	41	4	91.1%	31	14	68.9%
valley A	45	44	1	97.8%	43	2	95.6%
valley B	45	42	3	93.3%	39	6	86.7%
valley C	45	21	24	46.7%	8	37	17.8%
valley D	45	15	30	33.3%	0	45	0.0%
valley E	45	43	2	95.6%	25	20	55.6%
valley F	45	44	1	97.8%	41	4	91.1%

### Principal Component Analysis

**Figure 2:**
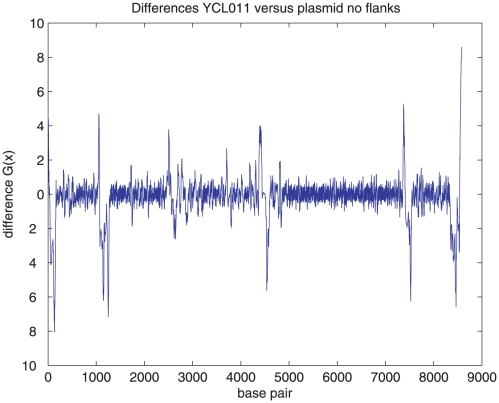
The difference profile of the YCL011 construct plasmid region is shown. This is the difference between the 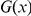 energy values for that construct and those for the plasmid alone, with no flanking region.

Because the ten profiles of Figure 1 are quite similar by casual inspection, we used the following procedure to isolate their differences. We regarded the profile for the plasmid alone, without flanking sequences, to be our reference, then found the difference between each profile and this reference by subtraction. This produced nine profiles, which we call the difference profiles. An example is given in Figure 2. In all cases the largest differences are concentrated near the valleys A–F, and at the boundaries of the plasmid.

We then performed a principal component analysis to capture the patterns of variation within these difference profiles for the plasmids that have been inserted at different positions. Principal component analysis takes a set of correlated variables (in this case difference profiles), and transforms them into a set of uncorrelated principal components. The transformed observations are called 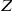-scores. Given a set of correlated variables 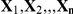, if the covariance matrix 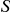


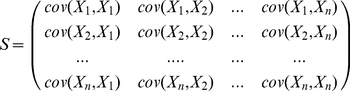
(1)may be diagonalized, (i.e. 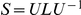 for orthonormal eigenvectors 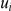,) then the Z-score for the 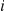 -th principal component may be derived as:
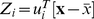
(2) where 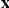 is a vector of observations from the original set of data. The theory behind principal component analysis is presented more fully in [24], for example.

Each principal component is a function of position along the insert sequence. Because we are analyzing nine difference profiles, this analysis produces a total of nine principal components. Then each of the nine difference profiles can be expressed uniquely as a specific linear combination of these nine functions, with appropriate values of the coefficients.

The eigenvalues and other information associated with the principal components derived from the difference profiles are presented in Table 2, ordered according to the percentage of variation that each captures. It is possible that some of the principal components with smaller eigenvalues may not be significant, but rather result essentially from random noise. The Bartlett test was developed specifically to address this issue [25]. This test determines whether the last 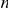 principal components are statistically indistinguishable from being equal, which they would be if they resulted from noise. This test showed that at least the first eight principal components found here from the difference profiles in fact are significant. The 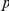 -values found by the Bartlett test also are given in Table 2.

**Table 2:** Results of the principle component analysis: eigenvalues, percentage of variation represented by each principle component and Bartlett test p-values.

Eigenvalue	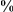 of variance	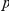 -value (Bartlett)
12.0546	83.00 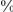	0
1.7093	11.77 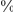	0
0.4919	3.39 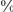	0
0.1771	1.22 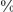	0
0.0438	0.30 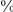	0
0.0220	0.15 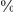	0
0.0183	0.13 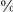	0
0.0044	0.03 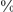	7.654E-105
0.0028	0.02 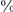	

The first principal component of the SIDD difference profiles captures the variation that these profiles have in common. As they are all similar, this first component captures the largest percentage of the observed variation, 83.3%. The second principal component captures 11.77% of the total variation, while the third component accounts for 3.39%. Because these first three components together account for 98.5% of the observed variation in the difference profiles, the analysis that follows focuses on them. While the lesser components are found by the Bartlett test still to be significant, their cumulative contributions are too small to be important in practice.

We have examined how the coefficients associated to the second and the third principal components for each profile correlate with the average G+C base composition of their flanks. For this purpose we considered the G+C content of both 5,000 bp flanks, averaged together, as this was previously found to correlate most closely with recombination rate [2]. The correlation between the coefficients for the second principal component and GC content was found to be 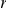  = 0.66, which the Pearson two tailed test finds to be significant at the 5% level. The coefficients for the third principle component were found not to be not significantly correlated with GC content. (Data not shown.) Thus, the second principal component appears to capture the effect of the sequence-averaged G+C content of the flanks.

### The Recombination Position Effect

To assess the recombination position effect, the recombination rates of all nine of our plasmid inserts have been previously experimentally measured [2], while the double strand break (DSB) frequencies are known for only six of them. However, for the six inserts for which both values are known there is an extremely strong correlation between these parameters. A linear regression analysis of DSBs versus recombination rates finds a positive correlation of 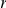  = 0.96. In what follows we concentrate on analyzing the recombination rate data because it is more complete, and because it is so closely correlated to DSB frequency that they are statistically virtually equivalent parameters.

**Figure 3:**
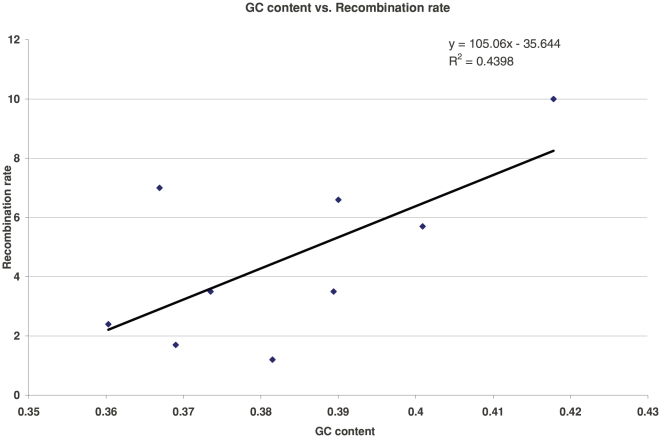
The G+C content of each of the nine flanking regions is plotted against their recombination rates. The regression line also is shown.

In their experimental study Petes and Merker [2] found that the G+C content of the flanking regions was significantly correlated with the measured recombination rate. When the flanking regions were each regarded as comprising 5,000 base pairs, the length used in the present study, they found a Pearson correlation coefficient of 0.75 between these parameters, and a Spearman correlation coefficient of 0.681. Figure 3 portrays the correlation between GC content and recombination rate. While these associations are suggestive, GC content itself cannot be the direct cause of the observed recombination position effect because it is a strictly local attribute. Instead, the observed context-dependent change in recombination rate must be due to some context-dependent consequence of the base composition of the flanks.

**Figure 4:**
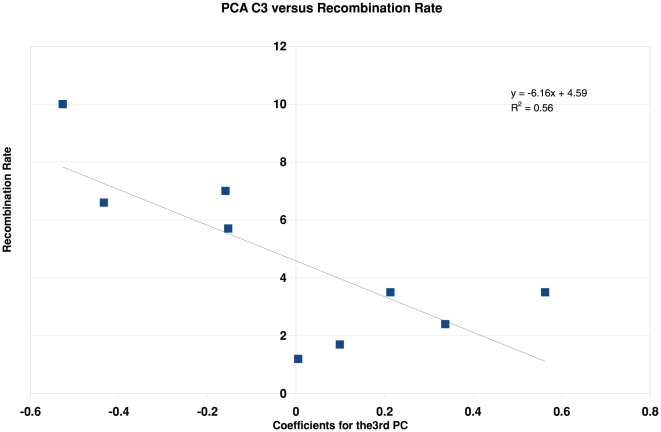
The correlation between the coefficients of the third principal component and the recombination rate.

The coefficients associated with the second principal component, described above, were found not to be significantly correlated with the recombination rates. However, the coefficients of the third principal component did correlate with recombination rate, with coefficient 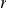 = −0.75. A graph of this data is shown in Figure 4. This is statistically significant at the 2% level, and is at least as strong a correlation as was found above for G+C content. This third principal component appears to capture a contribution of the SIDD properties to the recombination rate that is not due to the average G+C content of the flanks, because the coefficients of this component are not significantly correlated with that parameter. (Data not shown.) Instead, it may depend on higher resolution attributes involving the distribution of the GC base pairs in the flanks.

**Figure 5:**
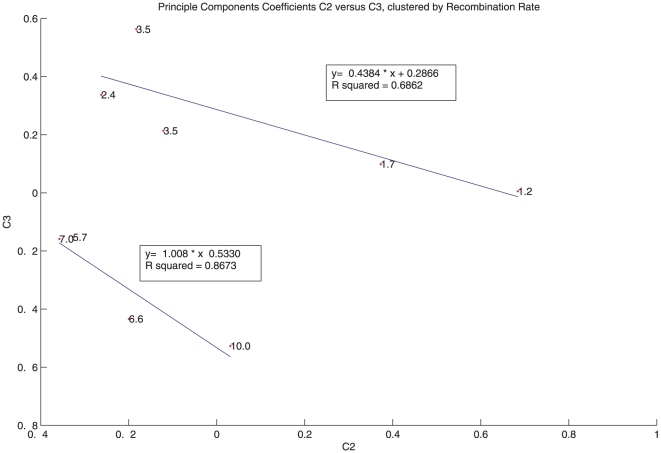
The recombination rate data forms two clusters in the plane of the second and third principal components. These coefficients are labeled C2 and C3, respectively. The recombination rate measured for each insert is written next to each point.

We next examined how both the second and third principal components, considered together, are associated with recombination rate. Figure 5 shows a graph of the coefficients of the second and third components, here called C2 and C3 respectively, that are associated with each insertion site. The nine points are labeled with the measured recombination rate in each case. Curiously, the data falls into two clusters, separated according to recombination rate. The four points with the highest recombination rates fall on one line with 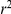  = 0.87, while the five points with the lowest recombination rates (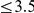) fall on a distinct line with 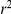 = 0.69.

**Figure 6:**
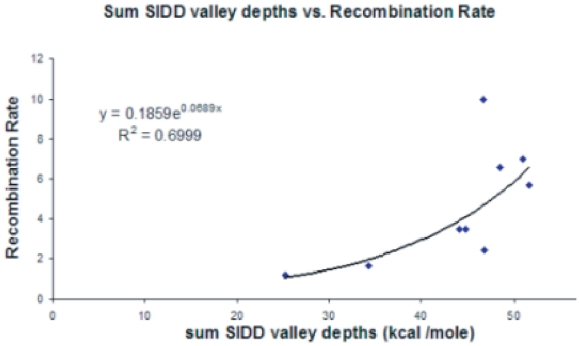
The scatter-plot is shown of the sums of the SIDD valley depths (in units of 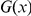 in kcal/mole) vs. the recombination rate for each of the nine plasmid inserts.

We also developed a second method to assess the relationship between recombination rates and SIDD properties. As above, we considered several measures of the 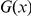 destabilization energies, including the lengths of the destabilized sites (i.e. valleys), the minimum value of 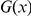 in each, and the area contained in each valley of the SIDD profile. In each case we evaluated the relationship between the SIDD measure and the recombination rate for each of the nine plasmid inserts. We found that the most informative measure was the sum of the minimum values of 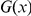 (i.e. the valley depths) over all six valleys. Figure 6 shows a scatter-plot of this SIDD measure versus recombination rate. An exponential model fits this data well, with correlation 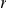  = 0.84. This is a substantially stronger correlation than that reported previously for G+C content. Because changes in the energy required to open a duplex region induce exponentially magnified changes in its equilibrium opening probability, the exponential fit found here is physically reasonable.

This aggregate SIDD measure is only very weakly correlated with the G+C content of the flanks, having 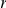 = 0.20 and p-value 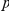 = 0.61. Since we only had nine observations to work with, we could not perform a multiple regression analysis to test which variable was the stronger predictor, or how they might covary. This would require many more observations (on the order of 100) in order to give reliable estimates.

## Discussion

In this paper we have investigated a possible new determinant of the recombination position effect. Although the recombination rate within an inserted plasmid correlates with the G+C content of its flanking regions, this effect cannot be directly due to this sequence attribute, but instead must derive from some related property that propagates into the interior of the plasmid. Here we investigate the possibility that this positional dependence of the recombination rate within the inserted plasmid arises through the changes of its superhelical destabilization properties that occur when it is placed in different locations. It is reasonable to surmise that recombination rates and double strand break frequencies would be sensitive to the extent to which the duplex is destabilized near the participating sites. Here we have presented statistical evidence to support this possibility.

First, we have shown that the destabilization profile of the plasmid insert is indeed significantly changed when it is placed at different genomic positions. These changes are substantially confined to the edge regions of the plasmid and to the six destabilized (SIDD) sites that occur within it. The SIDD sites that are closer to the edge of the plasmid are significantly changed somewhat more frequently than are the more interior sites, as would be expected from an effect that propagates from the flanks to the interior. The distribution of the SIDD energy 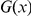 across the entire plasmid also has been shown to differ significantly between inserts in pair-wise tests.

These differences were examined more rigorously in the difference profiles, in which the SIDD profile of each insert sequence is subtracted from the SIDD profile of the circular plasmid alone. A principal component analysis was performed on these nine difference profiles. It found that at least the first eight principal components were significant. However, we concentrated on the first three components because they account for 98.5% of the variation in the data. We find that the coefficients of the second principal component are significantly correlated with the average G+C content of the flanks but not with the recombination rate, while those of the third component are significantly correlated with the recombination rate but not with the average flank G+C content. The statistical significance of the correlation of the third principal component with the recombination rate was at least as great as that previously found between recombination rate and average flank G+C content. This suggests that, although the G+C content of the flanks and SIDD properties both significantly affect recombination rate, they seem to do so in somewhat different ways. This is expected, as the SIDD properties would be affected also by the distribution of GC base pairs within the flanks, not just by their average G+C richness.

Finally, we found that an exponential function closely fits the recombination rate to the destabilization properties (sum of valley depths over the six SIDD sites), with correlation coefficient 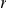 = 0.84. This is a significantly stronger correlation than that between recombination rate and G+C content. The correlation between this SIDD measure and G+C content was found to be only 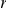 = 0.2, so that these two explanatory variables are not strongly correlated with each other. This reinforces the conclusion that these two explanatory variables seem to identify different influences of the flanking sequence.

The analyses presented here show that SIDD properties are statistically significant predictors of the level of the genomic instability of this plasmid, as measured by its recombination rate in different contexts. This suggests that the recombination position effect found by Borde et al. [1] may arise in part from the changes of stability that occur within superhelically stressed DNA sequences due to the influence of their flanking regions. If SIDD properties are involved in determining recombination rates, as our results suggest, this could explain a variety of other observations. These include the known correlation between recombination hotspots and promoter-containing intergenic regions, the effect of local binding proteins on hotspot activity, and the association of DSB formation with nuclease-hypersensitive sites [1], [2], [14].

Our results show that both SIDD measures and the G+C content of flanking regions correlate with recombination frequencies. However, because they do not correlate significantly with each other, they seem to identify different ways in which the flanking sequence affects the recombination rate. This suggests the possibility that these (along perhaps with other) attributes could be incorporated into a tool for predicting recombination rates of genomic regions based on both sequence and superhelical stress properties. Given sufficient data, it would be useful to develop a multiple regression model that simultaneously quantifies the contributions of both SIDD properties and G+C content to the recombination position effect. These matters remain for future investigation.

Other approaches have been used to analyze DNA duplex stability by using near-neighbor energetics to model the melting properties of linear DNA chains [26]–[28]. These methods are not expected to be useful in understanding the recombination position effect because melting energetics alone, without the coupling induced by superhelicity, is a strictly local, context-independent attribute. Thus any profile that only considers thermodynamic stability without superhelicity will give the same profile for the inserted pmj115 plasmid sequence, regardless of its context. This contrasts with the SIDD model, where the superhelical stresses couple together the behaviors of all base pairs that experience them. Thus the context dependence of the rate of DSB formation and the recombination rate, as found by Borde et al. [1] and by Petes and Merker [2], are much better described by the SIDD model than by any context-independent effect.

Here we have presented a method for statistically analyzing differences in the superhelical stress profiles of a fixed genomic sequence under different circumstances. This approach may be applicable in other situations where similar continuous parameter profiles of any type are to be compared. These could involve either placing a fixed DNA sequence in different genomic contexts, as here, or putting it under varying environmental or topological conditions, or comparing attributes of similar DNA sequences. For example, the viral vectors used in gene transfer are known to behave differently depending on their insertion sites [29], [30]. In lysogeny an infecting virus integrates its DNA into the genome of its host cell. Here also the behavior of the viral DNA can vary according to its genomic context. Similar issues arise for retroelement integration, which is known to occur preferentially at certain regions. A retroelement may contain a scaffold/matrix attachment region (S/MAR) that can induce site-dependent changes in the chromatin structure at its integration site, and thereby affect the regulation of host genes. Here identifying the S/MAR region within the retroelement and defining its role in modulating the regulation of nearby host genes becomes a problem of interest [31].

The approach developed here also could be useful when an experiment is carried out on the same sequence of DNA, but at various temperatures, salt concentrations and/or supercoiling densities, as in studies of DNA melting and of the effects of binding or drug interactions. For example, binding sites for the anti-cancer drug bizelesin are concentrated at matrix attachment regions [32] which are known to have high potential for supercoiling-induced duplex destabilization. In such cases, computational analysis of the genomic region(s) of interest may provide insight into the dynamics and mechanisms of activity of the agent. In all such examples it is important to be able to assess whether positional or environmental effects induce significant differences in the properties of the DNA.

An approach similar to the one developed here could prove useful for analyzing other types of sequence profiles. For example, Anselmi et al. [33] used thermodynamic methods to model nucleosome thermodynamic stability in terms of effective intrinsic curvature. Here a deformation energy profile is calculated by determining the energy cost required to deform each base pair in the sequence to the curvature that fits the crystal structure of the nucleosome. Again, Vologodskii and Frank-Kamenetskii [34] calculated differential melting profiles of DNA using the method of Fixman and Freire [35] and compared them to experimental results. The statistical method presented here could easily be applied to these cases.
